# E-cigarettes induce toxicological effects that can raise the cancer risk

**DOI:** 10.1038/s41598-017-02317-8

**Published:** 2017-05-17

**Authors:** Donatella Canistro, Fabio Vivarelli, Silvia Cirillo, Clara Babot Marquillas, Annamaria Buschini, Mirca Lazzaretti, Laura Marchi, Vladimiro Cardenia, Maria Teresa Rodriguez-Estrada, Maura Lodovici, Caterina Cipriani, Antonello Lorenzini, Eleonora Croco, Silvia Marchionni, Paola Franchi, Marco Lucarini, Vincenzo Longo, Clara Maria Della Croce, Andrea Vornoli, Annamaria Colacci, Monica Vaccari, Andrea Sapone, Moreno Paolini

**Affiliations:** 1 Department of Pharmacy and Biotechnology, Alma Mater Studiorum-University of Bologna, Via Irnerio 48, 40126 Bologna, Italy; 2 Department of Life Sciences, University of Parma, Parco Area delle Scienze 11A, 43124 Parma, Italy; 3 Inter-Departmental Centre for Agri-Food Industrial Research, Alma Mater Studiorum-University of Bologna, Via Quinto Bucci 336, 47521 Cesena, Italy; 4 Department of Agricultural and Food Sciences, Alma Mater Studiorum-Università di Bologna, Viale Fanin 40, 40127 Bologna, Italy; 5 Department of Neurofarba, Section of Pharmacology and Toxicology, University of Florence, Florence, Italy; 6 Department of Biomedical and Neuromotor Sciences, Alma Mater Studiorum-University of Bologna, Via Irnerio 48, 40126 Bologna, Italy; 7 Department of Chemistry “G. Ciamician”, Alma Mater Studiorum-University of Bologna, Via S. Giacomo 11, 40126 Bologna, Italy; 8 Department of Agricultural Biology and Biotechnology, CNR, Via Moruzzi 1, 56124 Pisa, Italy; 9 Center for Environmental Toxicology, Environmental Protection and Health Prevention Agency Emilia-Romagna Region (ER-EPA), Bologna, Italy

## Abstract

Electronic cigarettes (e-cigs) are devices designed to deliver nicotine in a vaping solution rather than smoke and without tobacco combustion. Perceived as a safer alternative to conventional cigarettes, e-cigs are aggressively marketed as lifestyle-choice consumables, thanks to few restrictions and a lack of regulatory guidelines. E-cigs have also gained popularity among never-smokers and teenagers, becoming an emergent public health issue. Despite the burgeoning worldwide consumption of e-cigs, their safety remains largely unproven and it is unknown whether these devices cause *in vivo* toxicological effects that could contribute to cancer. Here we demonstrate the co-mutagenic and cancer-initiating effects of e-cig vapour in a rat lung model. We found that e-cigs have a powerful booster effect on phase-I carcinogen-bioactivating enzymes, including activators of polycyclic aromatic hydrocarbons (PAHs), and increase oxygen free radical production and DNA oxidation to 8-hydroxy-2′-deoxyguanosine. Furthermore, we found that e-cigs damage DNA not only at chromosomal level in peripheral blood, such as strand breaks in leucocytes and micronuclei formation in reticulocytes, but also at gene level such as point mutations in urine. Our results demonstrate that exposure to e-cigs could endanger human health, particularly among younger more vulnerable consumers.

## Introduction

The lack of tobacco combustion, the most attractive feature of e-cigarettes (e-cigs), still allows smokers to inhale the aerosol in the same way as conventional cigarettes. E-cigs provide a copying mechanism for conditioned smoking by replacing some of the rituals associated with the automatic gestures of “regular” smoking^1^. Moreover, the possibility to use e-cigs in smoke-free places, the lack of specific regulations and the perceived potential for harm reduction^1^ make e-cigs very popular. For all these reasons, e-cigs are considered an alternative to tobacco cigarettes and an effective strategy to quit smoking. Contrary to the general belief that the lack of tobacco combustion typical of electronic nicotine-delivery systems avoids the production of harmful chemicals, the high temperature reached by e-cig solutions (>200 degrees Celsius)^2^ can generate dozens of toxic substances^3–5^, including tobacco-specific PAHs, nitrosamines, metals, carbonyl compounds such as acrolein and formaldehyde, which is classified as carcinogenic to humans (group 1, by the International Agency for Research on Cancer, IARC) and acetaldehyde, possibly carcinogenic (group 2B)^3^. Although e-cigs contain lower levels of these substances than tobacco cigarettes, these toxic mixtures have given rise to recent safety concerns^3–5^, stressing the need for appropriate toxicological data on these devices. The aim of the present study was to investigate several toxicological aspects associated with e-cig use including their mutagenic and co-mutagenic potential in a rat model.

## Results and Discussion

The volatile compounds (VOCs) disclosed by the GC/MS analysis on the e-cig aerosol were in agreement with the literature^3, 5, 6^. The main VOCs detected in our study were propylene glycol (PG), nicotine and vegetable glycerin (VG), together with other minor compounds and flavours (such as 1,2-propanediamine, methyl propionate, indole, propanoic acid 1-methylpropyl ester, acetol, 1-methoxy-2-propyl acetate, 3-hexen-1-ol, diacetyl and acrolein) (Table 1). In particular, heating of VG produces temperature-dependent amounts of hazardous aldehydes (such as formaldehyde, acetaldehyde and acrolein), due to thermal decomposition by free-radical dehydration of glycerol: formaldehyde, acetaldehyde and acrolein are formed at 600 °C, whereas acrolein is produced in some ionic environments at 350 °C^7^.

**Table 1:** Volatile compounds (VOCs) detected in the first and last treatment chambers during exposure to e-cig vapour.

	Chamber 1		Chamber 5		Statistical significance
	Mean	Standard deviation	Mean	Standard deviation	
1,2-Propanediamine	0.83	0.08	1.09	0.11	ns
Acrolein	0.02	0.00	0.03	0.02	ns
Indole	0.19	0.24	0.18	0.02	ns
Acetol*	0.07	0.03	0.07	0.00	ns
3-Hexen-1-ol*	0.05	0.00	0.06	0.02	ns
Diacetyl*	0.03	0.01	0.08	0.01	ns
Propylene glycol (PG)	87.71	1.03	88.66	0.19	ns
1-Methoxy-2-propyl acetate	0.07	0.04	0.05	0.01	ns
Methyl propionate*	0.20	0.01	0.21	0.06	ns
Propanoic acid, 1-methylpropyl ester	0.09	0.00	0.09	0.02	ns
Nicotine	6.36	0.62	6.54	0.18	ns
Glycerin (VG)	4.36	1.68	2.98	0.05	ns
PG/VG	21.80	8.63	29.80	0.43	ns

Values are expressed as percentage (%) of total peak area of VOCs; factorial analysis of variance (ANOVA) was performed to study the effect of exposure cycling on the formation of VOCs. Statistically different means were investigated (Tukey’s test, *P* < 0.05); *flavor compounds.

Since variables such as device brand, device wattage, resistive heating wire, nicotine concentration, PG/VG ratio and puff duration could significantly influence the emission of hazardous compounds (e.g. acrolein) and the rate at which nicotine is emitted per unit time^8^, we recorded the changes in the VOC profile throughout the exposure to e-cig vapour. No significant differences (*P* > 0.05) were found in the VOC composition of the different exposure chambers during animal treatment. This finding and the constant PG/VG ratio confirm that the same e-cig aerosol composition was supplied to the animals and no devices overheated. On the other hand, no trace of formaldehyde was detected, probably due to the procedure used to determine the VOC profile that entails derivatization steps, leading to selective determination of VOCs. However, no significant changes (*P* > 0.05) were detected in the amounts of acrolein (0.02–0.03% of total VOCs), VG or PG, suggesting that similar amounts of other aldehydes (not detected) were supplied throughout the treatment cycles, so the effects detected on the rats are related to all the compounds formed by e-liquid vaporization and present in the aerosol.

**Figure 1:**
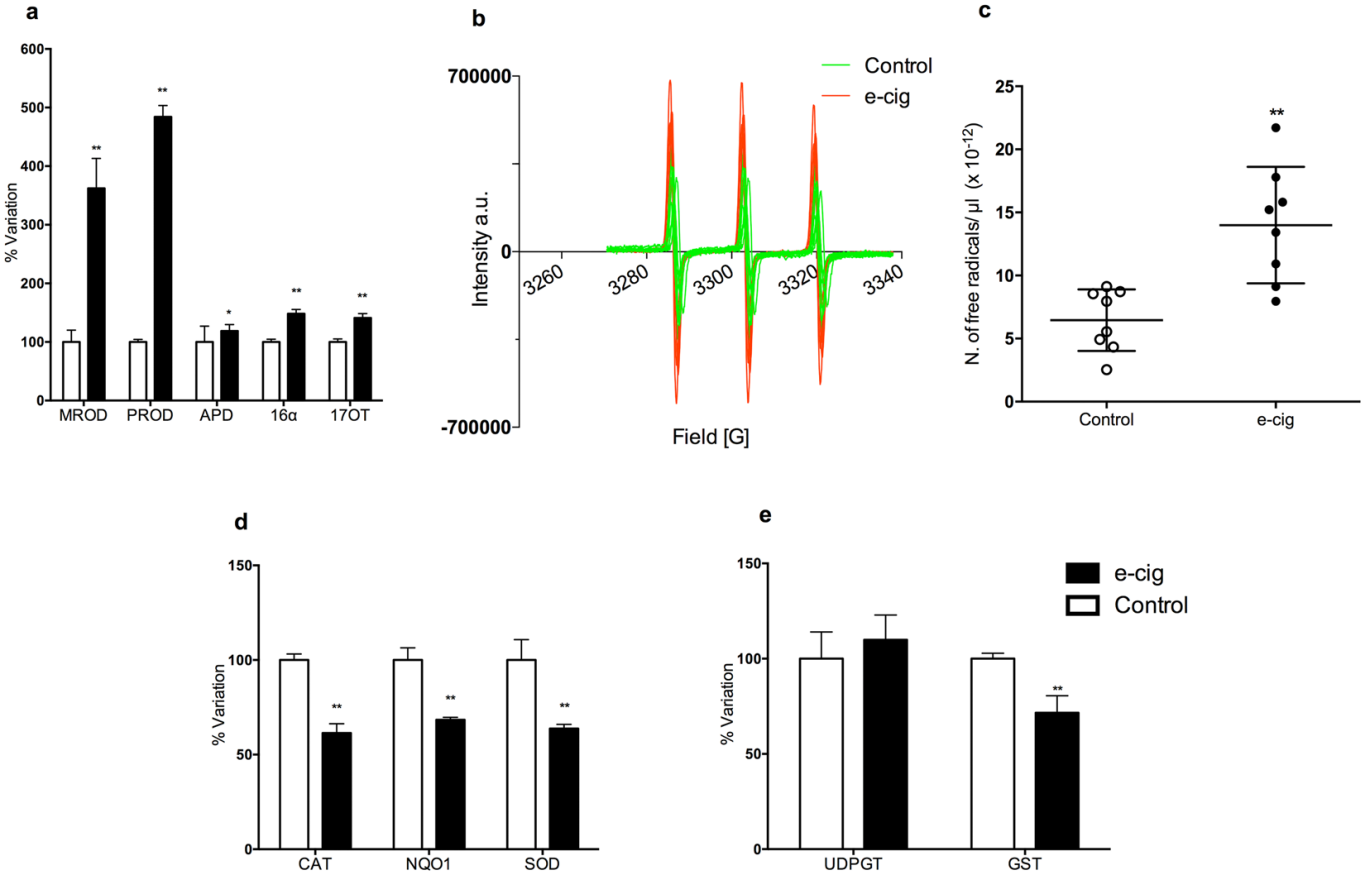
Metabolic/antioxidant enzymes and free radical yield in e-cig-exposed rat lung. (**a**) Cytochrome P450 (CYPs) is a superfamily of major isoenzymes involved in drug metabolism. CYP activities lead to the bioactivation of ubiquitous pre-mutagens and pre-carcinogens as well as ROS generation linked to their catalytic cycle. Data were obtained through enzymatic assays performed on microsomal lung fractions using several specific probes: MROD (CYP1A2-like) increased up to 262%, PROD (2B1/2) 384%, APD (3A, 1A, 2A, 2D) 19% *P* < 0.05, 16-α TOH (2B1/2C11) 48% (*P* < 0.01), 17-TOH (3A1) 41% (*P* < 0.01). (**b**) EPR spectra of nitroxide radicals observed in rat lung tissues in control samples (green spectra), and in e-cig vapour-treated samples (red spectra). (**c**) EPR intensity of the first spectral line of the observed nitroxide radicals (arbitrary units). (**d**) Antioxidant enzymes: CAT, NQO1 and SOD were reduced more than 32% (*P* < 0.01). (**e**) Transferases shown here are involved in the detoxifying step of xenobiotic metabolism making drugs or toxins more water-soluble. They also contribute to preserving DNA from adduct formation converting carcinogens into inactive or less toxic compounds: UDP-GT unchanged, GST 28% loss (*P* < 0.01). Each bar represents the means ± S.D. of ten measurements performed on ten rats, ^*^ *P* < 0.05, ^**^ *P* < 0.01, two-tailed *t*-test.

To explore whether e-cigs induce toxicological effects, such as those involving cytochrome P450 (CYP) changes^9^, we analysed the modulation of carcinogen-metabolizing enzymes in the lungs of rats exposed to e-cig vapour (see Methods). We observed a significant increase in CYP1A1/2 (activating, for example, polychlorinated biphenyls, aromatic amines, dioxins and PAHs), CYP2B1/2 (activating olefins and halogenated hydrocarbons), 2C11 (activating nitrosamines and mycotoxins) and CYP3A (activating hexamethyl phosphoramide and nitrosamines) documented by the sharp rise in the corresponding probes (Fig. 1a). Extrapolated to humans, the corresponding boosted CYP-linked monooxygenases would predispose a subject to an enhanced cancer risk from the widely bioactivated e-cig vapour procarcinogens associated with an increased risk of lung cancer with CYP induction and/or CYP polymorphisms^10^. The overproduction of reactive oxygen species (ROS) resulting from CYP induction is one of the well-documented ways in which CYP can play a key role in new cancer occurrence via a co-carcinogenesis mechanism^11^. Until the early 1990s, this phenomenon was initially associated only with induction of the CYP2E1 isoform. Then in 1996, we found that virtually all upregulated CYP isoforms can overproduce ROS^12^ primarily by uncoupling the CYP catalytic cycle.

We then used the electron paramagnetic resonance “EPR-radical probe” technique to evaluate the free radical content in lung. We found a significant increase in radical species yield in the lung (Fig. 1b,c). Our data are consistent with those recently reported by Sussan *et al*.^13^ showing e-cig vapour induced the development of oxidative stress in the lung. It is reasonable to hypothesize that the CYP induction found here, together with the free radicals present in the aerosol^13, 14^, contributed to the higher levels of ROS detected in exposed rats. Notably, we observed that the antioxidant enzymes catalase, DT-diaphorase and superoxide dismutase were all markedly reduced (Fig. 1d). Conversely, the conjugating phase II glutathione *S*-transferases, mainly involved in xenobiotic detoxification, were noticeable decreased, whereas UDP-glucuronyl-transferase was substantially unchanged (Fig. 1e). Thus, the reduced activity of antioxidant machinery, the free radicals reported to be present in vapour along with those generated by CYP induction found here can contribute to the inflammatory response^6, 13, 15^ and suggest an impairment of redox homeostasis in the lung.

**Figure 2:**
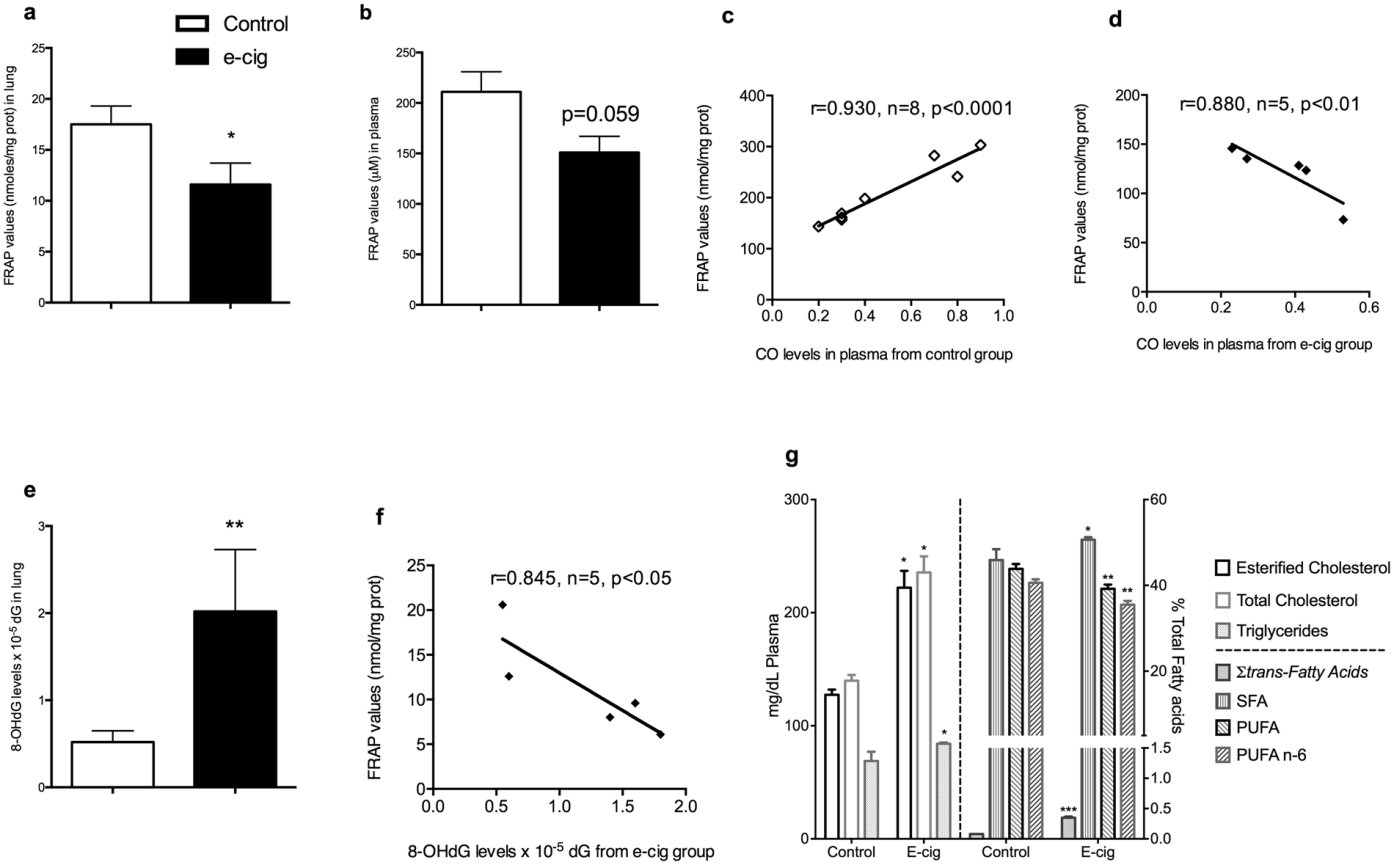
Systemic antioxidant capacity, oxidative DNA damage and lipidomics. (**a**) FRAP lung, loss >33% (*P* < 0.05). (**b**) FRAP plasma (*P* = 0.059). (**c**) FRAP plasma from e-cig group inversely correlated vs CO (r = 0.930, *P* < 0.001). (**d**) FRAP plasma from control group positively correlated vs CO (r = 0.880, *P* < 0.01). (**e**) 8-OHdG lung levels markedly increased ~288% (*P* < 0.01). (**f**) FRAP lung from e-cig group inversely correlated vs 8-OHdG (r = 0.845, *P* < 0.05). Data (n = 5 measurements per group) are expressed as means ± standard error of the mean (SEM), analysed by one-way analysis of variance (ANOVA) (**g**) *left side*, content of esterified cholesterol, total cholesterol and triglycerides (mg/dL) determined by GC/MS and GC/FID for qualitative and quantitative analysis, respectively, on Control and E-cig groups. *Right side*, sum of C18:1 *trans* isomers, saturated fatty acids (SFA), polyunsaturated fatty acids (PUFA) and PUFA n-6 series (PUFA n-6) in percentage (%) of total fatty acids determined by GC/FID on Control and E-cig groups. Each bar represents the means ± S.D. of ten measurements performed on ten ^*^ *P* < 0.05, ^**^ *P* < 0.01, ^***^ *P* < 0.001, two-tailed *t*-test.

To examine whether these phenomena affect the antioxidant power, we measured the systemic antioxidant capacity using the ferric reducing antioxidant power (FRAP) approach, finding a markedly reduced FRAP value in the lung (Fig. 2a). A similar trend was observed in plasma, even if statistical significance was not reached (*P* = 0.059) (Fig. 2b). Interestingly, plasma FRAP levels and measurement of carbonyl residues (CO) as biomarkers of oxidative injury to proteins were inversely correlated in e-cig vapour-exposed rats (Fig. 2c). In contrast, animals from the control group showed the opposite behaviour (Fig. 2d), indicating that control animals were able to increase their antioxidant capacity in relation to oxidative stress, while the lack of a protective antioxidant response in e-cig-exposed animals might explain the reduced FRAP level associated with CO formation. We also measured guanosine oxidation to 8-hydroxy-2′-deoxyguanosine (8-OHdG). 8-OHdG is one of the most extensively studied and abundant free radical-induced oxidative DNA lesions, which also correlates with mutagenesis in bacterial and mammalian cells^16^. Based on this evidence, 8-OHdG has been widely used as a biomarker to evaluate the load of oxidative stress and carcinogenesis^17^. We found that 8-OHdG markedly increased in the lungs of e-cig rats (Fig. 2e). This was supported by an inverse correlation between FRAP and 8-OHdG in lung tissue from exposed animals (Fig. 2f).

Insights into the redox imbalance also emerged from the study of the lipidome (Fig. 2g). The main lipid classes (free fatty acids, free cholesterol, esterified cholesterol and triglycerides) were determined by GC/MS. After e-cig aerosol exposure, the overall lipid composition of rat plasma was markedly affected with significant increases in the content of esterified cholesterol (EC), total cholesterol (TC) and triglycerides (TG) (*P* < 0.05) (Fig. 2g). These results agree with those reported by recent literature studies^18^ showing increased concentrations of TG, VLDL and TG/HDL ratios after four weeks’ intraperitoneal injection of nicotine and e-cig refill liquid containing nicotine in rats. Since the liver is the main organ responsible for cholesterol and lipoprotein synthesis, exposure to e-cig vapours might have affected rat liver function. On the other hand, the nicotine could have triggered the release of catecholamines and cortisol, in turn leading to activation of adenyl cyclase in adipose tissue and lipolysis of stored TG, with a subsequent increase in plasma VLDL and TG^19^. Tobacco smoking is known to lead to significantly higher serum concentrations of cholesterol and TG^20–22^. We did not explore the mechanism underlying this phenomenon, but nonetheless consider the finding noteworthy. In addition, we detected significant variations in the fatty acid composition of plasma: in particular, the sum of C18:1-*trans* isomers significantly (*P* < 0.001) increased probably due to the interaction of reactive VOCs generated by e-cig and plasma lipids. A significant increase in saturated fatty acids (SFA) was also found, whereas the content of polyunsaturated fatty acids (PUFA) and PUFA n-6 series noticeably decreased (Fig. 2g). Recently, Shen *et al*.^23^ demonstrated that alterations in cellular glycerophospholipid biosynthesis are an important consequence of e-cig vapour exposure due to enriched gene expression, and this could explain the fatty acid differences found here. However, in-depth histopathological investigation of diverse organs (such as liver) could yield more information on the lipidome changes found after e-cig aerosol exposure, and thereby shed more light on the underlying mechanisms involved.

**Figure 3:**
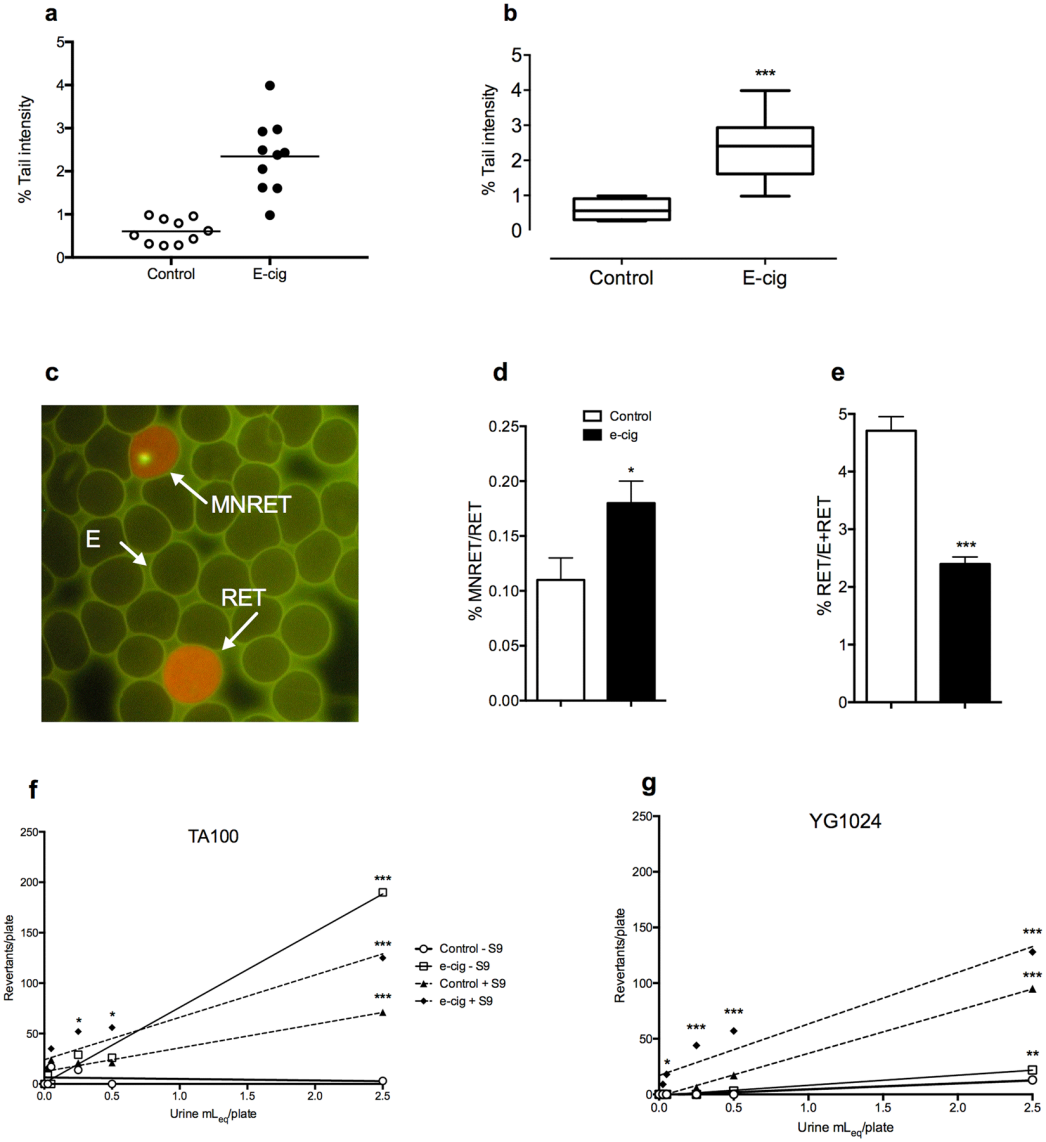
Genotoxicity of e-cig vapour. (**a**) Distribution of individual median TI% for the alkaline Comet assay. (**b**) Box-plot of TI%: primary DNA damage increase (*P* < 0.001). (**c**) Representative image of micronuclei (yellow MN-RET, orange RET, green erythrocytes (E). (**d**,**e**) MN-RET vs RET and RET vs E plus RET; MN-RET vs RET increased (*P* < 0.05); hematopoietic depression up to 50% loss, as RT fraction of total red blood cells, (*P* < 0.001). Error bars ± S.D. ^*^ *P* < 0.05; ^***^ *P* < 0.001 two-tailed *t*-test. (**f**,**g**) Urinary mutagenesis: TA100 and YG1024 S. typhimurium revertants/plate increased in a dose-dependent manner ± S9 mix. ^*^ *P* < 0.05; ^**^ *P* < 0.01; ^***^ *P* < 0.001 Bonferroni’s test).

To investigate the putative genotoxic potential of e-cig vapour, we considered various genetic endpoints at chromosomal and gene level in rat peripheral blood and urine, which served as body collectors of mutagenic metabolites. We observed that e-cigs produce extensive DNA damage in leukocytes measured as tail comet length of the fragmented DNA determined by single- and double-strand breaks (Fig. 3a,b). These data are in line with previous *in vitro* outcomes on HaCaT, UMSCC10B, and HN30 cell lines exposed to nicotine-containing and nicotine-free vapour extracts from two popular e-cig brands^24^. We also found that e-cig vapour determines an increase in the percentage of immature micronucleated reticulocytes (MN-RET) over normal reticulocyte RT (Fig. 3c,d). These results indicate that the mixture of chemical compounds generated by e-cigs leads to chromosome fragmentation and possibly damage to the mitotic spindle or centromeres. We observed a concomitant severe hematopoietic depression on exposed rats (Fig. 3e). Next, the urine of e-cig-exposed animals induced a dose-dependent increase in the number of S. typhimurium revertants in different strains. The highest sensitivity was shown by the TA100 strain (Fig. 3f), revealing base substitutions, and YG1024 (Fig. 3g), disclosing frame-shift mutations with an increased sensitivity to nitroarenes and aromatic amines. Mutant induction was affected by the S9 external metabolic activation system, suggesting both mutagenic and promutagenic metabolites in the urine.

Despite its shortcomings, the work presented here strongly raises the possibility that e-cig consumption under certain conditions leads to toxicological outcomes directly and indirectly damaging DNA in the rat. As shown in Table 1, our GC/MS analysis of the vapour was consistent with the literature^3^ confirming the presence, among others, of acrolein, toxic and mutagenic compounds^25^. However, our study currently precludes any cause-effect speculation, ascribing responsibility for the effects detected to the vapour as a whole rather than the single components. Our results should be construed as stemming from a preliminary study which was not conceived to replicate human vaping conditions, but to demonstrate if exposure to the chemical cocktail derived from e-cig liquid vaporization can result in toxicological injury.

As these detrimental phenomena are typically induced by conventional cigarettes^26–28^, the erroneous belief that e-cigs are safe should be retracted and suitable measures implemented to protect public health. Our study should be seen as the starting point for further investigations designed to confirm the harmful health impact of e-cigs, and a thorough analysis of their risk-benefit ratio, particularly after long-term exposure and under different usage conditions.

## Materials and Methods

### E-cigarette and liquid refills

The electronic device (e-cigarette) was composed of a 2.5 mL liquid tank in Pyrex glass and a rechargeable lithium battery (3.7 Volt EH IMR 18650; 2000 mAh), coupled with a dual coil atomizer (2 Ohm stainless steel resistance). The liquid used was purchased from BandZ S.r.l., (Pisa, Italy). The commercial brand is “Essential cloud, red fruit flavour”, 20 mL package, composition per 100 g of product: propylene glycol Ph.Eur., vegetable glycerine Ph.Eur., deionized water, flavours (“red fruits”), nicotine (18 mg/mL). Both e-cigarette and liquid refills were commercially available. The voltage was set at 5.5 V and the wattage was about 15 W.

### Preliminary conditions and chamber assessment

Several chemical analyses were conducted to determine the best conditions for experimental animal exposure. In particular, O_2_, N_2_ and CO_2_ were measured in the exposure chamber using an airtight syringe to establish a suitable O_2_/CO_2_, O_2_/N_2_ ratio and nicotine level to avoid interference with animal health during the experiment. Air was sampled with a Hamilton airtight syringe (30 mL), immediately transferred into a 5-mL capped vial and injected into GC/MS (QP-2010 Plus, Shimadzu, Japan) equipped with A RTX-WAX column (30 m, 0.25 mm i.d., 0.25 μm film thickness, Restek, USA), interfaced with a computerized system for data acquisition (Software GC–MS Solution V. 2.5, Shimadzu, Japan). The essential condition for the experiment starting point was a modest decrease in oxygen level (less than 5%) and a slightly higher CO_2_ level after the entire exposure time.

### Determination of total volatile compounds (VOCs)

Volatile compounds (VOCs) were extracted by headspace-solid phase microextraction (HS-SPME)^29^ and determined by GC–MS (QP-2010 Plus, Shimadzu, Japan), interfaced with a computerized data acquisition system (Software GC/MS Solution V. 2.5, Shimadzu, Japan), as reported by Cardenia *et al*.^30^ with a few minor modifications. An RTX-WAX column (30 m, 0.25 mm i.d., 0.25 μm film thickness, Restek, USA) and an SPME device with a fused-silica fiber (10 mm length) coated with a triphasic stationary phase (DVB/CAR/PDMS of 50/30 mm thickness), were used. The fiber was exposed to the chamber headspace after 17 s of puff (6 s on, 5 s off, 6 s on) for 1 min. Thereafter, the fiber was withdrawn into the needle and transferred to the injection port of the GC/MS system. The chamber headspace was sampled in three different positions (in triplicate) in the first and last chamber of the cycle exposure treatment. Samples were analysed under the same analytical conditions previously reported^14^. The acquisition and integration modes were Full Scan (TIC). Compounds were identified by comparing their mass spectra with those reported in the NIST08 (National Institute of Standards and Technology, Gaithersburg) library.

### Animal exposure

All experiments were carried out according to EU Directive 2010/63/EU. The protocol was approved by the University of Bologna Committee on the Ethics of Animal Experiments and by the Italian Ministry of Health (Permit number 26832015).

Male Sprague Dawley rats were purchased from ENVIGO RMS S.r.l. (San Pietro al Natisone, Udine, Italy) at 8 weeks of age. They were housed under a 12 h-light/12 h-dark cycle, 22 °C, 60% humidity, and fed ad libitum. After 5 days’ adaptation, the rats were randomly split into two groups: non-exposed as the control group and exposed (10 animals per group). The treatment group was exposed using a whole-body mode. The inhalation chamber consisted of a propylene box (38 × 26.5 × 19 cm) with a capacity of 19 L. The pump (0.18 kW; 1.4/1.6 A; 230 V; 50/60 Hz) was installed on one side of the box, while e-cigarette aerosol was puffed on the other. This mechanism generates airflow into the chamber. The chamber containing two animals at a time was not hermetically sealed and the two holes (e-cig and pump connection points) were left open. Animals were exposed in order to consume 1 mL/day of e-liquid containing 18 mg/mL of nicotine. One cycle of treatment consisted in 17 s puff (6 s on, 5 s off, 6 s on) followed by 20 min stop. During the experiment the e-cigarette voltage was set at 5.5. At the end of the cycle the animals were transferred to a clean chamber to begin the next cycle. Animals were submitted to 11 cycles/day for 5 consecutive days/week, and for 4 consecutive weeks.

Five additional rats were treated with mitomycin C (1 mg/kg, ip, single dose)^31^ to have positive controls for the micronucleus test. The animals were sacrificed no earlier than 24 h, but no later than 48 h after the treatment with mutagens, in order to collect tissues at the most appropriate timing to check the endpoints (micronuclei).

### Tissue collection

Rats were fasted 16 h prior to sacrifice. Animals were anesthetized by administering Zoletil 100 (100 mg/kg b.w.) and then sacrificed by decapitation according to the Ministerial procedures approved for the species. Lungs were collected and put in liquid nitrogen. Blood (caudal vein access) and 12 h urine (using metabolic cages) were also collected. Whole blood was collected for Comet assay and micronucleus test, while another fraction was decanted into anticoagulant tubes and centrifuged at 1,000 × *g* for 10 min to obtain plasma for the other parameter determinations (carbonyl residues (CO); ferric reducing ability of plasma (FRAP)).

### Subcellular fractions

Microsomes and cytosolic fraction were prepared as previously reported^32^.

### Xenobiotic phase-I/phase-II metabolism and antioxidant enzymes

All the assays have been described in detail elsewhere^33, 34^.

### Protein concentration

It was determined according to the method described by Lowry *et al*.^35^ using bovine serum albumin as standard and diluting microsomes 200 times and cytosol 1,000 times to provide a suitable protein concentration.

### Electronic paramagnetic resonance (EPR) measurements

Immediately before measurement, the frozen lung tissues were dissolved in a physiological solution containing the hydroxylamine “spin trap” (bis(1-hydroxy-2,2,6,6-tetramethyl-4-piperidinyl) decandioate dihydrochloride CAS no. 314726-62-0), and warmed for 5 min at 37 °C. The samples obtained were transferred and sealed in a calibrated capillary glass tube, which was placed inside the thermostated cavity (at room temperature) of a Bruker ESP 300 EPR spectrometer (Bruker Biospin S.r.l., Rheinstetten, Germany) equipped with a nuclear magnetic resonance gaussmeter for field calibration, a Bruker ER 033M FF-lock (Bruker Biospin S.r.l.) and a Hewlett-Packard 5350B microwave frequency counter (Hewlett Packard, Houston, TX, USA). The actual amount of solution analysed was chosen so as to cover the entire sensitive area of the instrument cavity. The spectra of the nitroxide radical, generated by the reaction of the probe with the radicals produced in the tissues, were then recorded using the following instrumental settings: modulation amplitude = 1.0 G; conversion time = 163.84 ms; time constant = 163.84 ms; modulation frequency 100 kHz; microwave power = 6.4 mW. The intensity of the first spectral line of the nitroxide (aN = 16.90 G and g = 2.0056) was used to obtain the relative amount of nitroxide in each examined samples. The calibration of the spectrometer response was done by using a known solution of TEMPO-coline in water and an ER 4119HS Bruker Marker Accessory as internal standard. The hydroxylamine probe was prepared as previously described^36, 37^.

### FRAP assay

This determination was performed in plasma and lung tissues. FRAP reagent (900 mL) containing 10 mM 2,4,6-tripyridyl-S-triazine in 40 mM HCl, 300 mM acetate buffer (pH 3.6) and 20 mM FeCl_3_ was added to 30 µL of plasma or supernatant tissue. The change in absorbance (at 593 nm) between the final reading and the blank was calculated for each sample and related to the absorbance of ferric standard solutions^38^.

### 8-hydro-2′-deoxyguanosine (8-OHdG) assay

This test was performed as previously reported^39^. Briefly, the lung was homogenized in 50 mM phosphate buffer solution (PBS) containing 0.1 M dithiothreitol and then centrifuged at 4 °C for 20 min at 2,000 × *g*. The pellets were resuspended and the DNA isolated. The purified DNA (about 30 µg) was hydrolysed with P1 nuclease (10 IU) and alkaline phosphatase (7 IU). The hydrolysed mixture was filtered using Micropure-EZ enzyme remover (Amicon, MA, USA) and 50 µL was injected into an HPLC apparatus. The nucleosides were separated by a C18 reverse-phase column (Supelco, 5 μm particle size, 0.46 cm I.D., 25 cm length). The 8-OHdG and 2 dG in the DNA were detected using an ESA Coulochem II electrochemical detector.

### Carbonyl residues

This determination was performed in the plasma and/or supernatant tissue^40^.

### Lipidomics analysis

Free fatty acids, esterified cholesterol, free cholesterol, triglycerides and total fatty acids were determined as reported in our previous work^41^ using GC/FID and GC/MS.

### Alkaline Comet assay

Directly withdrawn whole blood (10 µL) was added to 80 µL of 0.65% low melting agarose (LMA) in PBS. 5 µL of this solution were transferred onto degreased microscope slides previously dipped in 1% normal melting agarose using a 12-gel Comet assay unit (Severn Biotech Ltd, Worcestershire, UK). The agarose was allowed to set for 10 min. After agarose solidification, the slides were placed in lysing solution (2.5 M NaCl, 100 mM Na_2_EDTA, 10 mM Tris-HCl, 1% Triton X-100 and 10% DMSO, pH 10) in a Coplin jar at 4 °C overnight in the dark. Alkaline DNA unwinding was carried out in a gel electrophoresis chamber containing a freshly prepared buffer (1 mM Na_2_EDTA, 300 mM NaOH, pH 13) for 20 min and electrophoresis was performed in the same buffer for 20 min at 0.78 Vcm-1 and 300 mA. DNA unwinding and electrophoresis were performed in an ice-water bath. After electrophoresis the slides were washed in a neutralization buffer (0.4 M Tris-HCl, pH 7.5). All the steps described above were performed under a yellow light to minimize additional UV-induced DNA damage. After staining with 100 µL ethidium bromide (10 µg/mL), observations were made under a fluorescence microscope (Leica DMLS) equipped with an excitation filter BP 515–560 nm and a barrier filter LP 580 nm, using an image analysis system (Comet Assay IV– Perceptive Instruments Ltd, UK). DNA fragmentation tail (TI, tail intensity) provided representative data on the genotoxic effects. For each specimen 100 cells were analysed.

### Micronucleus test

After recoding to allow blinded analysis, smears of peripheral blood were methanol-fixed, stained with acridine orange (CAS Number 65-61-2, Sigma Aldrich) and analysed under fluorescence microscopy. For each animal 4,000 reticulocytes were scored to determine micronuclei frequency and 4,000 erythrocytes were scored to determine the ratio between reticulocytes and the sum of reticulocytes and erythrocytes. As positive controls, 1 mg/kg b.w. of mitomycin was administered i.p. as described above^31^.

### Ames test

Urine extracts were dissolved in DMSO (25 mLeq urine/mL) and tested with the Ames test in triplicate at increasing concentrations (0.025–2.500 mLeq urine /plate) using Salmonella typhimurium TA100 and YG1024 strains, with and without microsomal activation (S9 mix) to detect indirect and direct mutagenic compounds, respectively. Positive controls were 2-aminofluorene (2-AF; 5 µg/plate) for both strains with S9 and hycanthone (HY; 50 µg/plate) for both strains without S9. DMSO was tested as negative control. The Ames test data are reported as means of three independent replicates of revertants/plate^42^.

### Statistical analysis

Where not differentially specified, data are expressed as means ± standard deviation (S.D.) of ten measurements on ten rats for each studied group. Data from the study on volatile compounds (VOCs) and those obtained from the preliminary tests to set the experimental conditions of the exposure chamber were analysed using Tukey’s test. Results from xenobiotic metabolizing enzymes and antioxidant enzymes were analysed using the two-tailed unpaired t-test with the multiple-comparison post-hoc analysis (Holm-Sedak). Data from FRAP assay 8-OHdG, and CO (n = 5 measurements per group), were analysed using the two-tailed unpaired t-test. The micronucleus test was statistically analysed using the two-tailed unpaired t-test. Tukey’s test was used to analyse data from the lipidomics study. For the Comet assay and Ames test the distributions of the variables were preliminarily assessed by means of the one-sample Kolmogorov-Smirnov test, and parametric statistical tests were applied to normally distributed variables. The mean values from the repeated experiments were used in a one-way analysis of variance (ANOVA). If significant F-values (*P* < 0.05) were obtained, post hoc Student’s t test (Bonferroni’s version) was conducted for pairwise comparison. *P* values < 0.05 were considered statistically significant.
